# Evaluation of Programmed Death Ligand-1 (PD-L1) and B-cell Lymphoma 2 (BCL2) protein expression using immunohistochemistry in colorectal carcinoma patients from Anbar Governorate

**DOI:** 10.1186/s43046-026-00409-z

**Published:** 2026-09-04

**Authors:** Akram Baraa, Hekmat Ahmed, Mohammed Mohammed, Ahmed Abed

**Affiliations:** 1 College of Nursing, University of Al Maarif, Ramadi, Iraq; 2 College of Health and Medical Technology, University of Al Maarif, Ramadi, Iraq; 3 College of Medicine, University of Anbar, Ramadi, Iraq; 4 Generel Directorate Education of Anbar, Ministry of Education, Ramadi, Iraq

**Keywords:** PD-L1, Bcl‑2, Colorectal carcinoma, Immunohistochemistry, Expression

## Abstract

**Background:**

Colorectal carcinoma is among the most prevalent malignancies worldwide. PD-L1 is an immune-checkpoint ligand that can contribute to tumor immune evasion whereas BCL2 is an anti-apoptotic protein involved in cell-survival pathways, with its mutation frequently observed in various human cancers. This study aimed to evaluate the prognostic significance of immunohistochemically identified PD-L1 and BCL2 proteins in colorectal carcinoma.

**Materials:**

Sixty paraffin-embedded colorectal carcinoma tissue specimens were subjected to immunohistochemistry (IHC) staining for PD-L1 and BCL2 expression, which was analyzed using the DAKO scoring system (0: 0–4%, 1: 5–24%, 2: 25–49%, 3: 50–74%, 4: 75–100%).

**Result:**

PD-L1 protein expression was detected in all patients, with immunoreactivity noted in 33.3% (score 3), 31.7% (score 2), 20.0% (score 4), and 15.0% (score 1), with score 0 cases showing no detectable immunoreactivity Similarly, Bcl-2 protein expression was identified in all colorectal carcinoma patients, with immunoreactivity observed in 38.3% (score 3), 25.0% (score 2), 20.0% (score 4), and 16.7% (score 1), while no detectable immunoreactivity was seen in score 0 cases.

**Conclusions:**

These findings highlight the ubiquitous expression of PD-L1 and Bcl-2 in colorectal carcinoma and their potential role as prognostic biomarkers, providing insights into their relevance in cancer progression and therapeutic response.

## Introduction

Colorectal cancer (CRC) persists as a formidable public health issue, ranking among the foremost contributors to cancer-associated morbidity and mortality globally [1]. With the progression of knowledge regarding the molecular and immunological frameworks that underlie CRC, the tumor microenvironment has been recognized as a pivotal domain of investigation. This microenvironment encompasses a multifaceted interaction of tumor cells, immune cells, and molecular markers that significantly affect cancer advancement, metastasis, and patient prognoses [2]. Among these markers, Programmed Death Ligand-1 (PD-L1) and B-cell lymphoma 2 (Bcl-2) have attained notable attention due to their respective functions in immune evasion and apoptosis resistance. The examination of their expression via immunohistochemistry (IHC) provides crucial insights into their prognostic and therapeutic relevance in CRC [3].

This study focuses on CRC patients located in Anbar Governorate, Iraq, a region noted for its scarcity of cancer research and a rising prevalence of CRC. The assessment of PD-L1 and Bcl-2 expression within this cohort can yield localized understanding of CRC pathogenesis, facilitating the advancement of targeted therapeutic strategies and personalized medicine methodologies. This introduction delineates the roles of PD-L1 and Bcl-2 in CRC, the applicability of IHC in the identification of these markers, and the importance of this investigation within the framework of Anbar Governorate.

### Programmed Death Ligand-1 (PD-L1) in CRC

PD-L1 constitutes an immune checkpoint protein that is expressed on a variety of cellular types, including neoplastic cells. Through its interaction with the Programmed Death-1 (PD-1) receptor located on T-lymphocytes, PD-L1 effectively inhibits immune responses, allowing neoplastic cells to escape immune detection [4]. In colorectal cancer (CRC), the expression levels of PD-L1 have been correlated with numerous clinicopathological characteristics, such as tumor aggressiveness, lymph node metastasis, and microsatellite instability (MSI) status [5].

Immunohistochemical analyses have revealed that the expression of PD-L1 in CRC is heterogeneous, displaying variability between neoplastic and stromal cells [6]. Elevated PD-L1 expression has been associated with MSI-high tumors, which are distinguished by a heightened mutational burden and increased infiltration of immune cells [7]. These observations accentuate the potential of PD-L1 as a biomarker for forecasting responses to immune checkpoint inhibitors (ICIs), such as pembrolizumab, which has demonstrated efficacy in MSI-high CRC [8].

Beyond its involvement in immune evasion, the expression of PD-L1 has been investigated as a prognostic indicator in CRC. Although certain studies indicate a relationship between high PD-L1 expression and unfavorable prognosis, others propose a more favorable outcome in specific subpopulations, such as MSI-high patients [9]. These contradictory findings underscore the necessity for additional research to elucidate the prognostic significance of PD-L1 in CRC, particularly in populations that have been inadequately studied, such as those residing in Anbar Governorate.

### B-cell Lymphoma 2 (Bcl-2) in CRC

Bcl-2 is recognized as an anti-apoptotic protein that modulates cell survival by obstructing the mitochondrial pathway of apoptosis [10]. The dysregulation of apoptotic processes is a defining characteristic of cancer, with the overexpression of Bcl-2 being implicated in tumor progression and therapeutic resistance [2]. In CRC, Bcl-2 expression has been linked to various clinicopathological features, including tumor differentiation, stage, and response to chemotherapeutic agents [11].

Immunohistochemical investigations have shown that Bcl-2 expression in CRC is frequently inversely correlated with tumor grade and stage, with elevated expression levels observed in well-differentiated and early-stage tumors [12]. This trend suggests that Bcl-2 may serve a protective function during the early phases of CRC, potentially by stabilizing the cellular microenvironment. However, in more advanced stages, the overexpression of Bcl-2 may contribute to chemoresistance by impeding apoptosis induced by therapeutic interventions [13].

The dualistic nature of Bcl-2 in CRC emphasizes its intricate role as a biomarker. While its expression may signify a favorable prognosis in the initial stages of the disease, its involvement in therapeutic resistance underscores the imperative for targeted strategies aimed at overcoming Bcl-2-mediated chemoresistance. The combination of Bcl-2 inhibitors, such as venetoclax, with conventional chemotherapy regimens has demonstrated potential in preclinical investigations [14], thereby necessitating further exploration in clinical contexts.

### Immunohistochemistry in biomarker detection

Immunohistochemistry (IHC) constitutes a widely employed methodology for the detection of protein expression within tissue specimens. This technique presents the distinct advantage of spatial localization, thereby enabling researchers to evaluate the distribution of biomarkers across tumor and stromal compartments [15]. In the context of colorectal cancer (CRC), IHC has played a pivotal role in elucidating the expression patterns of PD-L1 and BCL2, thereby enriching our understanding of their implications in tumor biology and clinical outcomes [16].

The efficacy of IHC in discerning PD-L1 expression is thoroughly substantiated, with numerous studies employing diverse antibodies and scoring systems to assess its expression in both tumor and immune cell populations [17]. Nevertheless, the absence of standardization in IHC protocols and their interpretation engenders challenges, resulting in discrepancies in reported findings. Correspondingly, IHC investigations of Bcl-2 expression have unveiled its heterogeneous distribution within CRC tissues, thereby underscoring the necessity for standardized methodologies to facilitate accurate evaluations [18].

Despite these obstacles, IHC persists as an invaluable instrument for biomarker research, particularly in resource-constrained environments such as the Anbar Governorate. The relatively modest cost and accessibility of IHC render it an optimal technique for the examination of PD-L1 and BCL2 expressions in CRC patients hailing from this region.

### Significance of research in Anbar Governorate

Research pertaining to colorectal cancer in Iraq, inclusive of the Anbar Governorate, remains sparse, with the majority of investigations concentrating on epidemiological and clinical dimensions [19]. The assessment of molecular and immunological markers, including PD-L1 and BCL2, is imperative for comprehending the pathogenesis of CRC within this demographic. Variables such as genetic susceptibility, environmental exposures, and dietary patterns may exert influence on the expression of these markers, thus necessitating localized investigative efforts.

This study aspires to bridge the existing knowledge void by examining PD-L1 and Bcl-2 expressions in CRC patients from Anbar Governorate through the application of IHC. By correlating these expressions with clinicopathological attributes, the study endeavors to identify potential prognostic indicators and therapeutic targets. The outcomes of this research could significantly contribute to the formulation of personalized medicine strategies, thereby enhancing CRC management within this underserved region.

### Patients study group

#### Tissue biopsy

A total of sixty tissue biopsy specimens from patients diagnosed with colorectal carcinoma were obtained from AL-Ramadi Teaching Hospital. The expression of Programmed Death Ligand-1 (PD-L1) and B cell lymphoma (BCL2) proteins was evaluated utilizing the immunohistochemistry technique. To ascertain the prognostic significance of PD-L1 and BCL2 in colorectal carcinoma, the expression levels of these proteins were meticulously analyzed in paraffin-embedded tissue sections derived from sixty colorectal carcinoma specimens via immunohistochemistry.

#### Patients and control groups

The study encompassed sixty male patients diagnosed with colorectal carcinoma through histopathological biopsy et al.-Ramadi General Teaching Hospital during the diagnostic timeframe from April 2022 to January 2024. The Patient’s group was exclusively composed of sixty male individuals, with ages ranging from 21 to 85 years. Furthermore, the control specimens consisted of ten histologically normal colorectal tissue samples obtained from areas adjacent to the tumors of patients undergoing surgical resection. The tissues were confirmed to be free of dysplasia or carcinoma by histopathological examination. These samples were not obtained from healthy volunteers. Their use was approved by the institutional ethical committee and informed consent was obtained (as applicable).

#### Method of immunohistochemistry

The immunohistochemical analysis was conducted on tissue biopsy samples obtained from colorectal carcinoma under the methodology described by [20], The deparaffinization process of the slides involved subjecting them to xylene treatment (twice) and a series of graduated alcohol dilutions (100% twice, followed by 95%, 70%, and 50%), concluding with an incubation in distilled water for ten minutes.

The slides were subjected to de-masking through incubation in a target retrieval solution, specifically Citrate Buffer at pH 9, for PD-L1 and B cell lymphoma (BCL2) for one hour at a temperature of 97 °C in a water bath. Following antigen retrieval, the slides were permitted to cool for twenty minutes at room temperature. Subsequently, the periphery surrounding the samples was delineated using a liquid blocker, Papen, to prevent the dispersion of materials beyond the samples during the immunohistochemical process. The slides were positioned in staining racks, and the cooled antigen retrieval solution was applied to prevent sample desiccation.

Each slide was washed once with a washing buffer. A peroxidase-blocking reagent was administered to all slides and incubated for ten minutes. The utilization of HRP-conjugated antibodies may induce elevated nonspecific background staining; therefore, this nonspecific background can be considerably diminished by pretreating cells/tissues with hydrogen peroxide before the incubation with HRP-conjugated antibodies. The slides were subsequently washed with the washing buffer, and a blocking reagent (antibody diluents) was applied for thirty minutes to mitigate excess nonspecific protein binding and reduce background formation. The slides were again washed with the washing buffer. The optimal dilution of each primary antibody was then applied to the slides, which were incubated for sixty minutes. Following this incubation, the slides were thoroughly washed with washing buffer. Incubations devoid of specific antibodies served as negative controls, while reference slides were designated as positive controls. Horseradish peroxide was introduced and incubated for forty minutes before washing with the washing buffer.

The chromogen 3,3-Diaminobenzidine was added and allowed to incubate for ten minutes. The slides were subsequently washed vigorously twice. Gill’s Hematoxylin and Eosin Solution were applied as a counterstain for four minutes, after which the slides were vigorously washed with water. The dehydration process for the slides was conducted, followed by their subsequent mounting utilizing a DPX mounting medium within the LEICA CV5020 mounting apparatus. The stained slides were allowed to air dry at ambient temperature within a fume hood for one hour. The stained slides were evaluated collaboratively with a certified pathologist employing a light microscope. The pathologist's expertise facilitated the determination of cut-off values for all antibodies utilized in the investigation. The cut-off values for PD-L1 and B cell lymphoma (BCL2) were established following the DAKO scoring system, which categorizes as follows: 0 (0–4%), 1 (5–24%), 2 (25–49%), 3 (50–74%), or 4 (75–100%) [21].

## Results

### The grading of colorectal carcinoma patients encompassed within this study

**Fig. 1:**
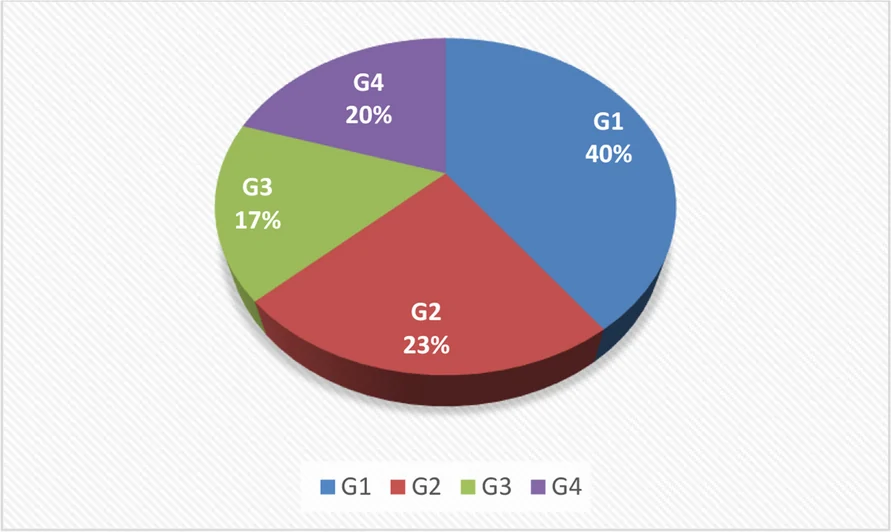
Percentage of the cancer grade in the colorectal carcinoma patients

The analyzed samples were as follows: G1 (Grade) represented the predominant category with a total of 24 cases (40%) of patients, succeeded by G2, which comprised the second largest group with 14 cases (23%) of patients, followed by G4, which constituted the third category with 12 cases (20%) of samples, and G3, which exhibited an equivalent count of 10 cases (17%) for each, representing the smallest cohort of patients, as illustrated in Fig. 1. These study descried the distribution of tumor grades in the study cohort.

### Stage of colorectal carcinoma patients

**Fig. 2:**
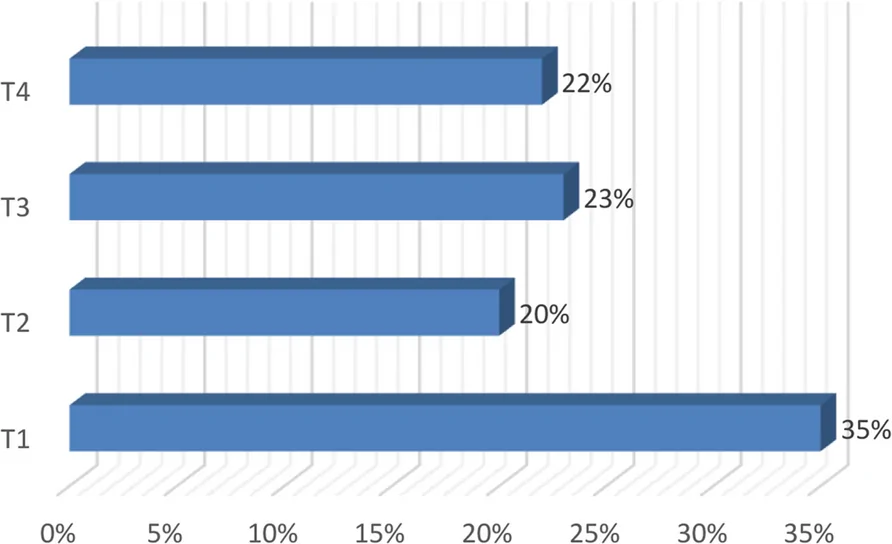
Percentages of the colorectal carcinoma stage in colorectal carcinoma patients

In relation to the stratification of malignant colorectal carcinoma, T1 (stage 1) accounts for the predominant proportion, encompassing 21 cases (35%), succeeded by T3 (stage 3) with 14 cases (23%). T4 represents the third category in frequency with 13 cases (22%), while T2 emerges as the least represented group, comprising 12 cases (20%) within the sampled population, as illustrated in Fig. 2.

### Distribution of colorectal carcinoma according to the age of patients

**Fig. 3:**
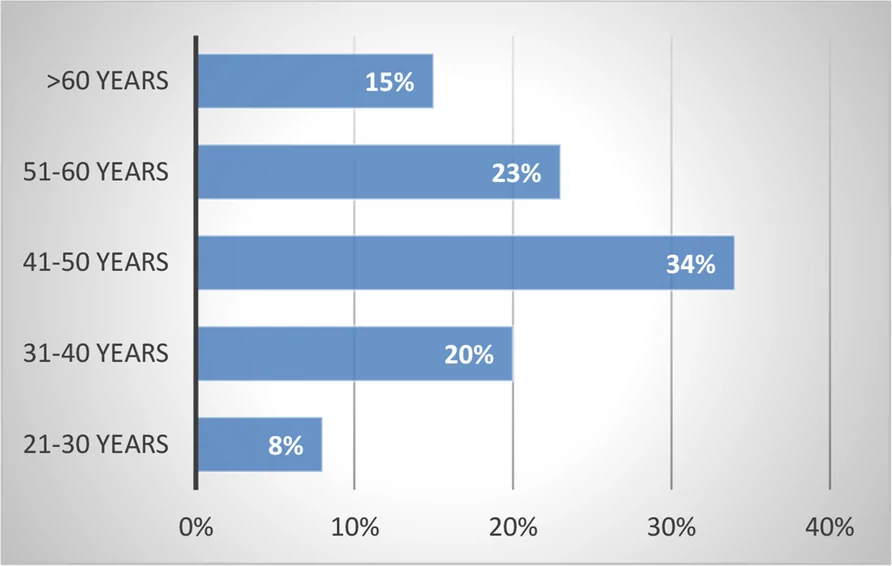
The age frequency of colorectal carcinoma patients according to the age of intervals

The distribution of study subjects diagnosed with colorectal carcinoma was systematically categorized into fifth groups based on age demographics. Patients situated within the fifth decade of life (aged 41 – 50 years) represented the most substantial segment, comprising (*n* = 20;34%) of the total study cohort, followed by the cohort in the sixth decade of life (aged 51 – 60 years) (*n* = 14; 23%). The subsequent cohort consisted of patients in the fourth decade of life (aged 31 – 40 years) (*n* = 12;20%), The subsequent cohort consisted of patients in the seventh decade of life (aged over 60 years) (*n* = 9;15%), while the remaining cohort encompassed individuals in the third decade of life (aged 21 – 30 years) (*n* = 5;8%), as delineated in Fig. 3.

### Results immunohistochemistry

#### Distribution of colorectal carcinoma according to the scoring of Bcl-2

**Fig. 4:**
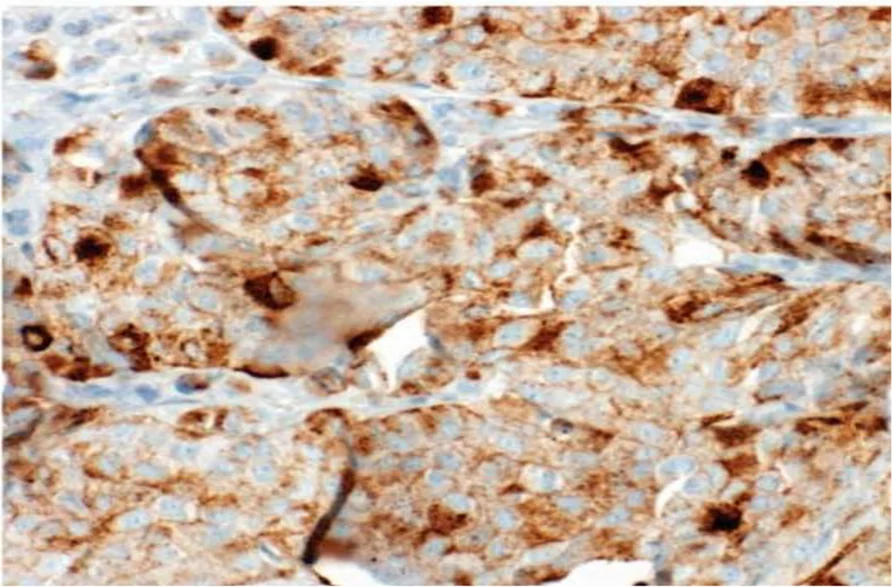
Representative Bcl-2 Immunohistochemically Staining in colorectal carcinoma; Tumor cells show cytoplasmic brown DAB immunoreactivity with hematoxylin counterstaining (blue); The image represents a high staining score (score 4; 75–100% Positive tumor cells). Original magnification (100X)

**Fig. 5:**
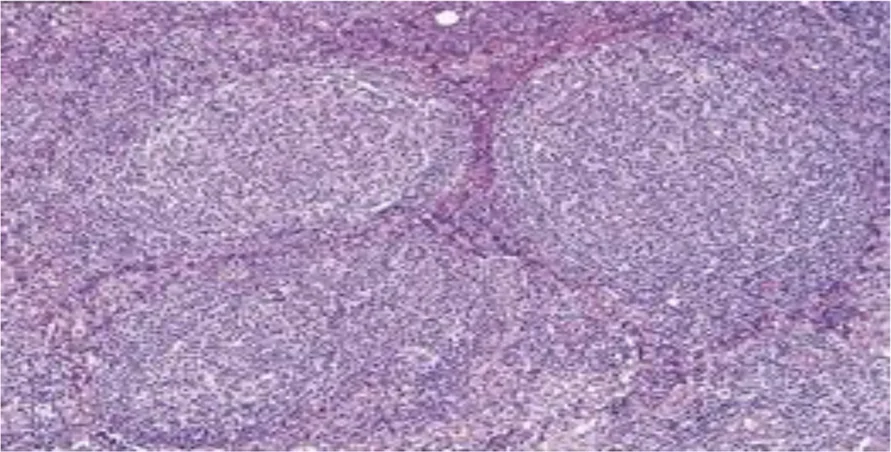
Representative Bcl-2 Immunohistochemically staining in the normal adjacent colorectal tissue. the tissue shows no detectable Bcl-2 immunoreactivity, corresponding to score 0 (0–4% positive cells). Brown staining is absent or limited to nonspecific background. hematoxylin counterstain; original magnification (100X)

The expression of Bcl-2 protein was thoroughly evaluated in all patients diagnosed with colorectal carcinoma. The results showed immunoreactivity in 23 cases (38.3%) with a score of 3, 15 cases (25.0%) with a score of 2, 12 cases (20.0%) with a score of 4, and 10 cases (16.7%) with a score of 1. A score of 0 indicated no detectable immunoreactivity. The detailed findings are presented in Table 1 and illustrated in Figs. 4 and 5.

**Table 1:** Frequency of Bcl-2 proteins in colorectal carcinoma patients and controls

Bcl-2 Score	Colorectal Carcinoma		Control	
	**Count**	**Percentage**	**Count**	**Percentage**
Score 0	0	0.0%	9	90%
Score 1	10	16.7%	1	10%
Score 2	15	25.0%	0	0%
Score 3	23	38.3%	0	0%
Score 4	12	20.0%	0	0%
Total	60	100%	10	100%

#### Distribution of colorectal carcinoma according to the scoring of PD-L1

**Fig. 6:**
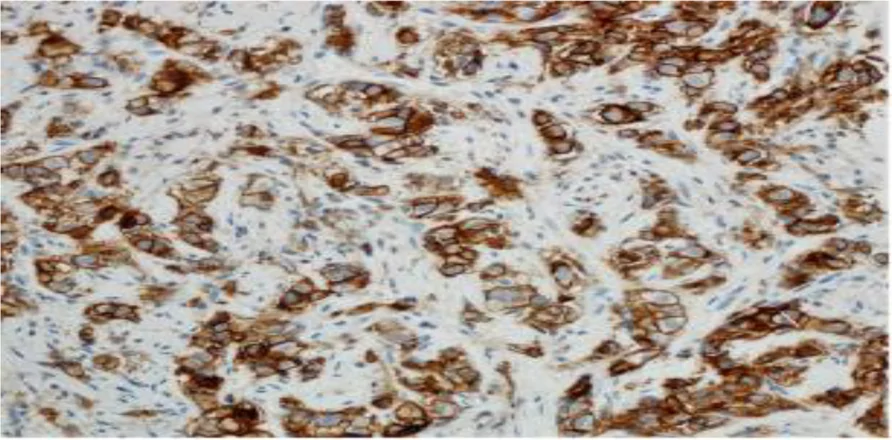
Representative PD-L1 immunohistochemical staining in colorectal adenocarcinoma. Malignant glandular epithelial cells show strong membranous and cytoplasmic brown DAB immunoreactivity (indicated by the black arrow) with hematoxylin counterstaining (blue). The image demonstrates a high staining score (score 4; 75–100% positive tumor cells). Original magnification: 100 ×

**Fig. 7:**
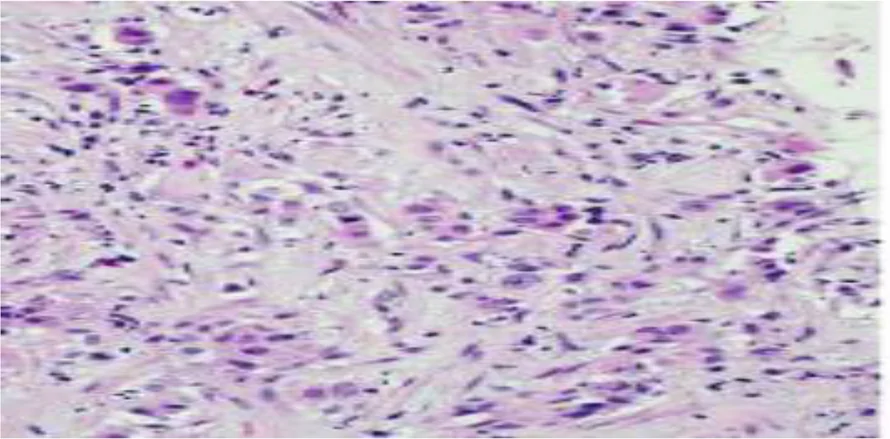
Representative PD-L1 Immunohistochemically staining in the normal adjacent colorectal tissue. the tissue shows no detectable PD-L1 immunoreactivity, corresponding to score 0 (0–4% positive cells). Brown staining is absent or limited to nonspecific background. hematoxylin counterstain; original magnification (100X)

The PD-L1 protein expression was similarly evaluated in all colorectal carcinoma patients, demonstrating immunoreactivity in 20 cases (33.3%) with a score of 3, 19 cases (31.7%) with a score of 2, 12 cases (20.0%) with a score of 4, and 9 cases (15.0%) with a score of 1, while a score of 0 denoted no detectable immunoreactivity, as depicted in Table 2 and Figs. 6 and 7.

**Table 2:** Frequency of PD-L1 proteins in colorectal carcinoma patients and controls

PD-L1 score	colorectal carcinoma		Control	
	**Count**	**Percentage**	**Count**	**Percentage**
Score 0	0	0%	8	80%
Score 1	9	15.0%	2	20%
Score 2	19	31.7%	0	0%
Score 3	20	33.3%	0	0%
Score 4	12	20.0%	0	0%
Total	60	100%	10	100%

## Discussion

The BCL2 oncogene is recognized as a significant inhibitor of apoptosis, which may facilitate the accumulation and dissemination of cells harboring genetic mutations [9]. It exhibits a close association with the process of carcinogenesis in the majority of malignant diseases. In the current investigation, the expression of Bcl-2 within colorectal carcinomas was identified utilizing immunohistochemistry (IHC) and was correlated with various clinicopathological parameters to evaluate the potential utility of Bcl-2 expression in the diagnostic and prognostic assessment of colorectal carcinoma [13]. The BCL2 gene encodes a 26-kD protein that functions to obstruct apoptosis and inhibit programmed cell death. Its mutation is frequently observed as a genetic alteration in human malignancies. Programmed death-ligand 1 (PD-L1), also referred to as cluster of differentiation 274 (CD274) or B7 homolog 1 (B7-H1), is a protein encoded in humans by the CD274 gene. Its expression can be readily detected through immunohistochemistry (IHC) [22].

The interaction between programmed cell death receptor 1 (PD-1) and programmed death ligand 1 (PD-L1) is essential for suppressing the immune mechanisms that allow cancer cells to evade antitumor immunity. Immunotherapy that employs checkpoint inhibitors is an emerging treatment option for various types of cancer. One such example is the use of anti-PD-1/PD-L1 therapies [23].

PD-L1 is mainly found on the surface of tumor cells and antigen-presenting cells in various solid tumors. This includes squamous cell carcinoma of the head and neck, melanoma, and carcinomas of the brain, thyroid, thymus, esophagus, lung, breast, gastrointestinal tract, colorectum, liver, pancreas, kidney, adrenal cortex, bladder, urothelium, ovary, and skin [24]. In the tumor microenvironment, the expression of PD-L1 on tumor cells and other tumor-promoting cells is activated by two mechanisms: a constitutive mechanism and an induced mechanism. Both mechanisms depend on two binding sites of IRF-1 [22]. For instance, in BRAFV600-mutated melanoma, the expression of PD-L1 results from the adaptive response of cancer cells to immune attacks stimulated by cytokines or may arise from a constitutive expression due to oncogenic processes [25]. PD-L1 is seldom expressed in normal tissues but can be induced at tumor sites, rendering the PD-L1 pathway distinctly different from other coinhibitory pathways, [4] suggesting that the selective expression of PD-L1 may correlate with the clinical outcomes of cancer patients and may serve as a targeted approach for antitumor therapy.

### Study limitations

The study has some limitations: First, the sample size was relatively small and only male patients were included from a single institution in Anbar Governorate, thus compromising the power and generalizability of the conclusions. Second, the retrospective design of tissues lacked follow-up for survival, recurrence, treatment response and cause-specific mortality, and therefore the prognostic and predictive value of PD-L1 and BCL2 was not established. Third, the clinicopathological data was sparse, and there were no data on lymph-node status, distant metastasis, tumor sidedness, mismatch-repair/microsatellite-instability (MSI) status, molecular alterations or treatment regimens. Fourth, interobserver variation, heterogeneity of the tumors, tissue fixation, staining protocol, and antibody clone can influence the immunohistochemically scoring. Lastly, there were limited numbers and sources of the control specimens, so caution should be used in comparing carcinoma and control specimens. Such restrictions need to be overcome in larger prospective multicenter trials with validated antibodies, uniform scoring systems and clinically annotated follow-up information.

## Conclusion

Immunoreactivity for the PD-L1 and BCL2 was observed in all cases of colorectal cancer collected from male patients in the Anbar Governorate, with varying staining scores. These findings demonstrate the presence and distribution of these proteins in the examined tumors. The present data, however, do not provide prognostic/predictive information about PD-L1 and BCL2, as the study did not have survival follow-up, treatment-response data or extensive clinicopathological correlations. Further prospective studies are warranted that include treatment-response assessment, size of the tumors, status of lymph nodes, status of metastases, mismatch-repair/microsatellite-instability status, and survival.

## Data Availability

No datasets were generated or analysed during the current study.
